# Human Case of *Bartonella alsatica* Lymphadenitis 

**DOI:** 10.3201/eid1412.080757

**Published:** 2008-12

**Authors:** Emmanouil Angelakis, Hubert Lepidi, Atbir Canel, Patrick Rispal, Françoise Perraudeau, Isabelle Barre, Jean-Marc Rolain, Didier Raoult

**Affiliations:** Université de la Méditerranée, Marseille, France (E. Angelakis, H. Lepidi, J.-M. Rolain, D. Raoult); Centre Hospitalier d’Agen, Agen, France (A. Canel, P. Rispal, F. Perraudeau, I. Barre)

**Keywords:** Bartonella alsatica, lymphadenopathy, cat-scratch disease, letter

**To the Editor:** Lymph node enlargement is a common medical problem that is usually caused by bacterial, viral, fungal, or protozoal agents (*1*). Malignancies or lymphoproliferative diseases are often found, especially in elderly patients (*1*). *Bartonella henselae,* the main causative agent of cat-scratch disease (CSD), appears to be the most common organism responsible for lymphadenopathy in adults and children (*1*). CSD has also been rarely associated with *B*. *quintana* (*2*). Recently, the epidemiology of *B. quintana* as an emerging source of human infection has changed because it has been isolated from the dental pulp of a domestic cat (*3*). Feral cats have also been found to be infected by *B*. *quintana* (*4*). We report a human case of *B. alsatica* lymphadenopathy.

A 79-year-old woman came to a hospital in Agen, France, in February 2008 with a large painless axillary mass that she had noticed 10 days earlier. She reported that ≈1 month earlier she was scratched on her finger while butchering a wild rabbit. On examination, she did not have any other specific findings. Blood cell counts and levels of liver enzymes were normal. A large necrotic lymph node was surgically removed the next day. Her condition was treated with doxycycline (200 mg) for 3 weeks.

Our laboratory received a fragment of the lymph node of the patient and a portion of the rabbit that had been cooked, boiled as a terrine, and stored in a freezer at –20°C in the home of the patient. DNA was extracted from these specimens by using a QIAamp Tissue Kit (QIAGEN, Hilden, Germany). The DNA was used as a template in 3 described PCRs specific for a portion of the *B*. *alsatica* 16S–23S intergenic spacer (ITS) region, *ftsZ* gene, and 16S rDNA (*5*). All results for the lymph node were positive for *B*. *alsatica*, and amplification products of the expected size were obtained from this extract. Sequences obtained shared 100% similarity with the corresponding 16S rDNA, ITS region, and *ftsZ* gene fragment of *B. alsatica*. However, the terrine specimen was negative for 16S rDNA, the ITS region, and the *ftsZ* gene. All negative controls showed typical results. *B*. *alsatica* have not been tested or found in our laboratory for several years.

*B. quintana* subsp. Oklahoma, *B. henselae* subsp. Houston (ATCC 49882), *B. vinsonii* subsp. *berkhoffi* (URBVAIE25), *B. vinsonii* subsp. *arupensis* (ATCC 700727), and *B. alsatica* (CIP 105477 T) strains were used for immunofluorescence and Western blotting assays (*5*). A serum sample taken at admission was negative for *B*. *alsatica* by immunofluorescence assay. This result was accepted because serologic results may be negative during the onset of the disease (*6*). Western blotting with *Bartonella* spp. antigens (*5*) was positive for *B. alsatica* and after adsorption, only *B. alsatica* antigens retained all antibodies (Appendix Figure, panel **A**).

Formalin-fixed, paraffin-embedded tissue specimens (3-μm thick) were stained with hematoxylin and eosin. Microscopic examination showed that the normal architecture of the lymph node was destroyed. Histologic changes were dominated by large irregular stellate or round granulomas with central neutrophil-rich necrosis (Appendix Figure, panel **B**). Granulomas were composed mainly of macrophages, whereas neutrophils in the necrotic areas were fragmented. These granulomas with abscess formation were similar to those described in CSD. Warthin-Starry staining showed bacteria in the necrotic center of the granulomas (Appendix Figure, panel **C**).

Immunohistologic staining was used to demonstrate *B. alsatica* in the lymph node. Immunohistochemical analysis was performed by using a monoclonal antibody against *B. alsatica* with an immunoperoxidase kit previously described (*7*). Briefly, after deparaffinization, the tissue section was incubated with polyclonal-specific antibody to *B. alsatica* (*8*) diluted 1:1,000 in phosphate-buffered saline. Immunodetection was performed with biotinylated immunoglobulins, peroxidase-labeled streptavidin (HistoStain Plus Kit; Zymed, Montrouge, France), and amino-ethyl-carbazole as substrate. Slides were counterstained with Mayer hematoxylin for 10 min. Location of bacteria was superimposable on that in the Warthin-Starry–stained specimens, and clusters of microorganisms were seen in the inflammatory areas (Appendix Figure, panel **D**).

We report lymphadenitis caused by *B. alsatica*. Our finding was confirmed by molecular, serologic, and staining methods. *Bartonella* spp. are zoonotic agents that infect erythrocytes of mammals in which they cause chronic bacteremia (*9*). *B. alsatica* was first identified in 1999 in Alsace, France, as an agent of bacteremia in healthy wild rabbits (*10*). However, in 2006, interest in *B. alsatica* increased when it was considered to be a human pathogen because it caused blood-culture–negative endocarditis in a patient who had contacts with rabbits (*5*). The present case confirms that *B. alsatica* could be a human pathogen, especially in persons who live in contact with rabbits and should be considered a cause of lymphadenopathy.

## Supplementary Material

Appendix Figure A) Western blotting analysis of lymph node specimen from the patient before 1) and after 2) cross-adsorption with Bartonella alsatica. Lane 1, B. quintana; lane 2, B. henselae; lane 3, B. elizabethae; lane 4, B. vinsonii subsp. berkhoffii; lane 5, B. alsatica. B) Characteristic histologic change in the lymph node with B. alsatica infection. Shown is an inflammatory granulomatous process with central microabscess surrounded by a ring of macrophages and rare giant cells (hematoxylin and eosin stain, original magnification ×100). C) Bacteria (arrow) in an abscess formation mixed with necrotic debris (Warthin-Starry silver stain, original magnification ×1,000). D) Immunohistochemical detection of B. alsatica (arrow) in lymph node pulp with an extracellular distribution (polyclonal antibody and hematoxylin counterstain, original magnification ×400).
