# Supplementary figures and images for: Human Case of Bartonella alsatica Lymphadenitis
Source: Emerg Infect Dis. 2008 Dec;14(12):1951–3. doi: 10.3201/eid1412.080757 (PMC2634634; doi:10.3201/eid1412.080757)

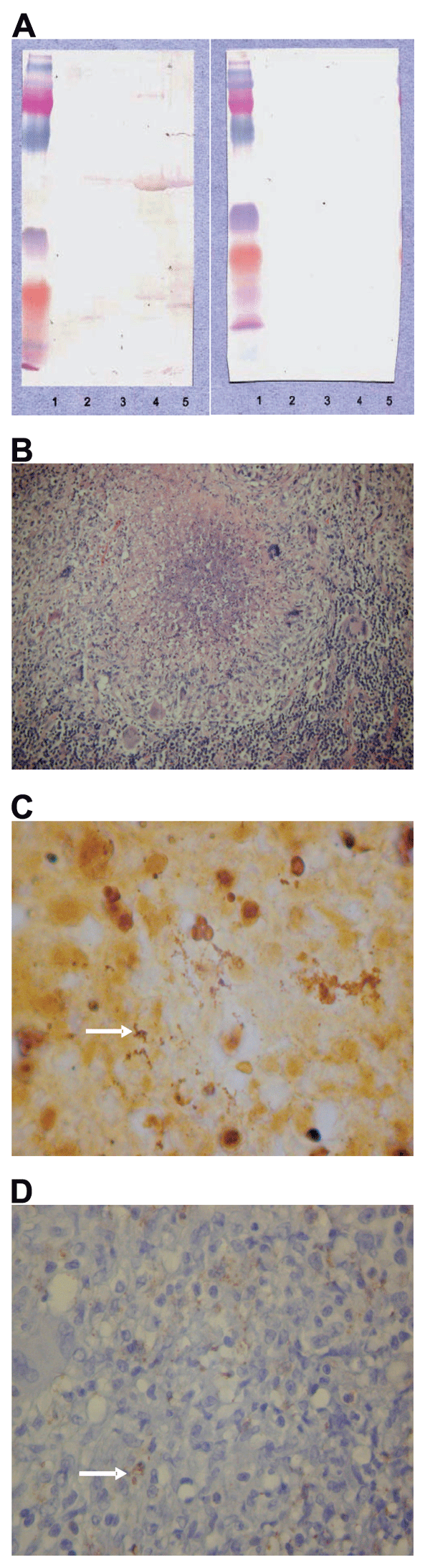

Supplement: Appendix Figure — A) Western blotting analysis of lymph node specimen from the patient before 1) and after 2) cross-adsorption with Bartonella alsatica. Lane 1, B. quintana; lane 2, B. henselae; lane 3, B. elizabethae; lane 4, B. vinsonii subsp. berkhoffii; lane 5, B. alsatica. B) Characteristic histologic change in the lymph node with B. alsatica infection. Shown is an inflammatory granulomatous process with central microabscess surrounded by a ring of macrophages and rare giant cells (hematoxylin and eosin stain, original magnification ×100). C) Bacteria (arrow) in an abscess formation mixed with necrotic debris (Warthin-Starry silver stain, original magnification ×1,000). D) Immunohistochemical detection of B. alsatica (arrow) in lymph node pulp with an extracellular distribution (polyclonal antibody and hematoxylin counterstain, original magnification ×400). [file 08-0757_app-s1.gif]
